# Supplementary figures and images for: Analysis and pollution evaluation of heavy metal content in soil of the Yellow River Wetland Reserve in Henan
Source: PeerJ. 2023 Dec 14;11:e16454. doi: 10.7717/peerj.16454 (PMC10725677; doi:10.7717/peerj.16454)

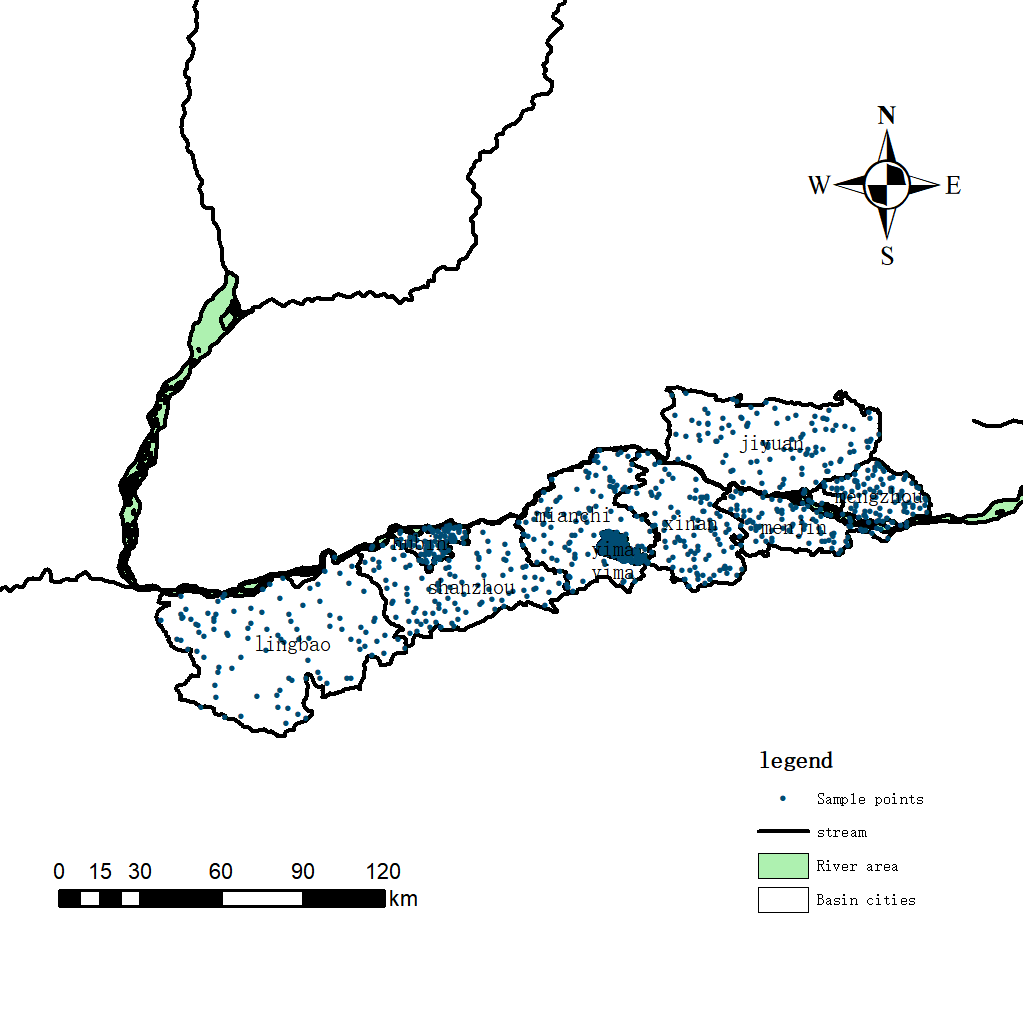

Supplement: Supplemental Information 1 [file peerj-11-16454-s001.zip › peerj-89657-peerj-89657-original_material_and_basic_data/1 (1).png]

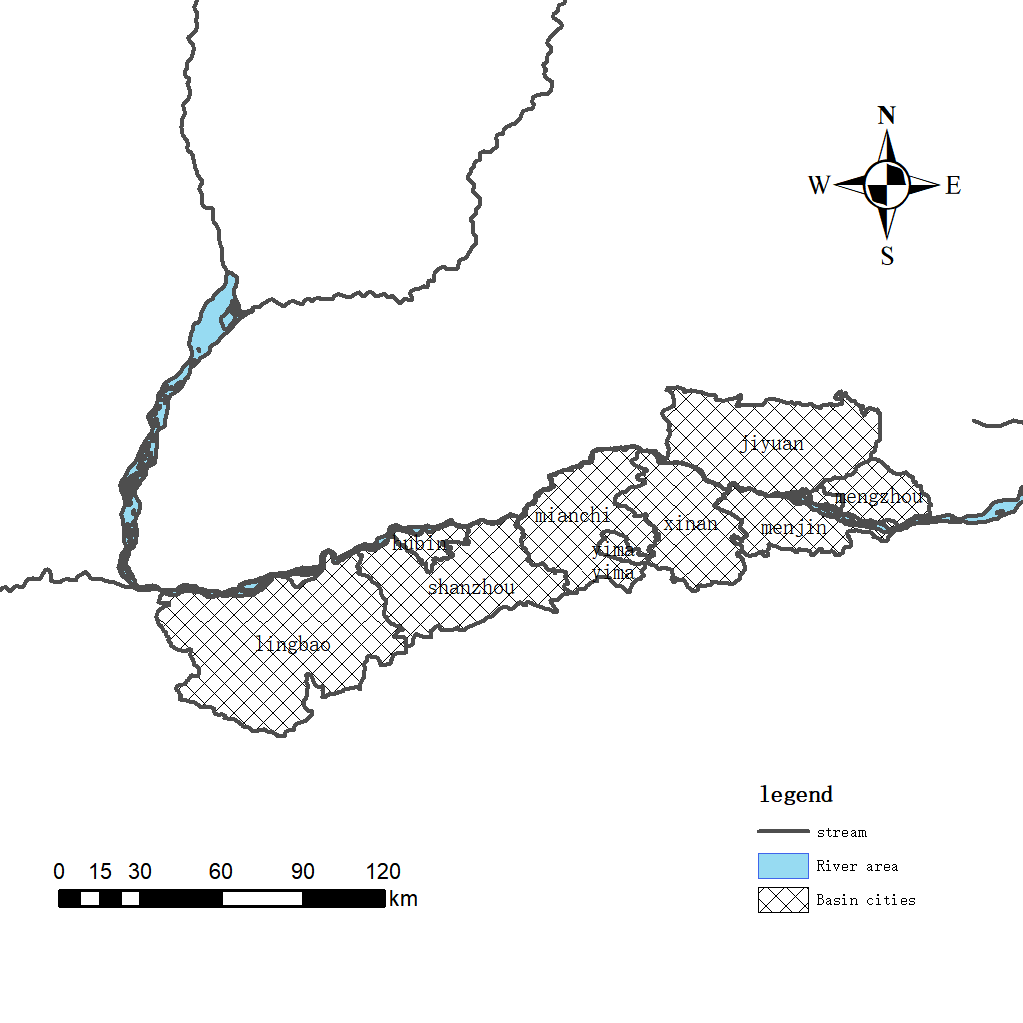

Supplement: Supplemental Information 1 [file peerj-11-16454-s001.zip › peerj-89657-peerj-89657-original_material_and_basic_data/1 (2).png]

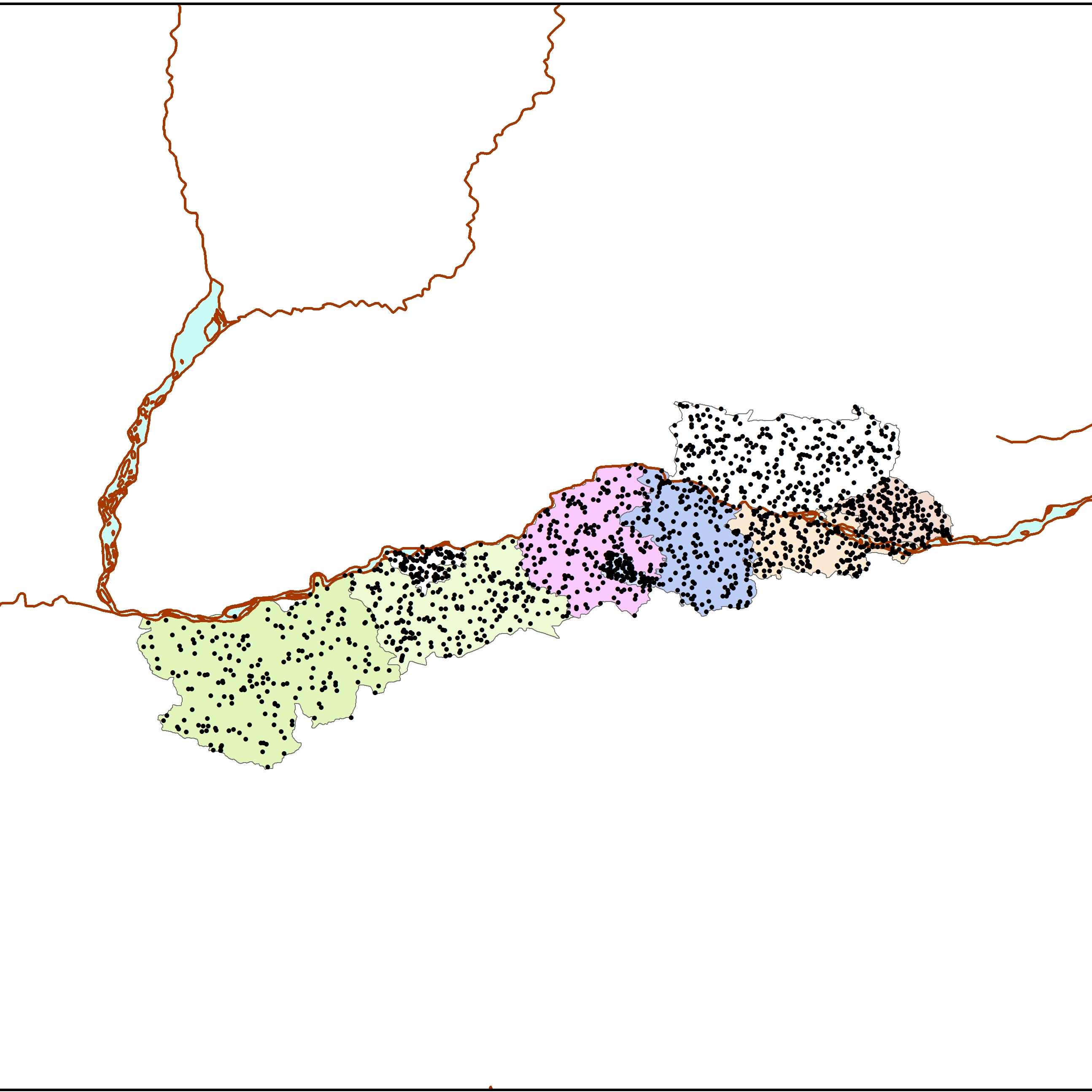

Supplement: Supplemental Information 1 [file peerj-11-16454-s001.zip › peerj-89657-peerj-89657-original_material_and_basic_data/1 (3).png]

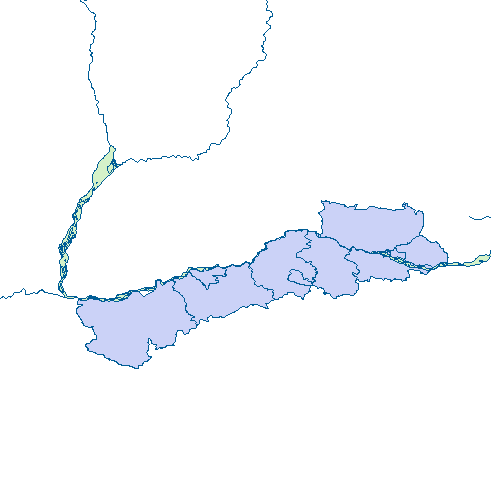

Supplement: Supplemental Information 1 [file peerj-11-16454-s001.zip › peerj-89657-peerj-89657-original_material_and_basic_data/1 (4).png]

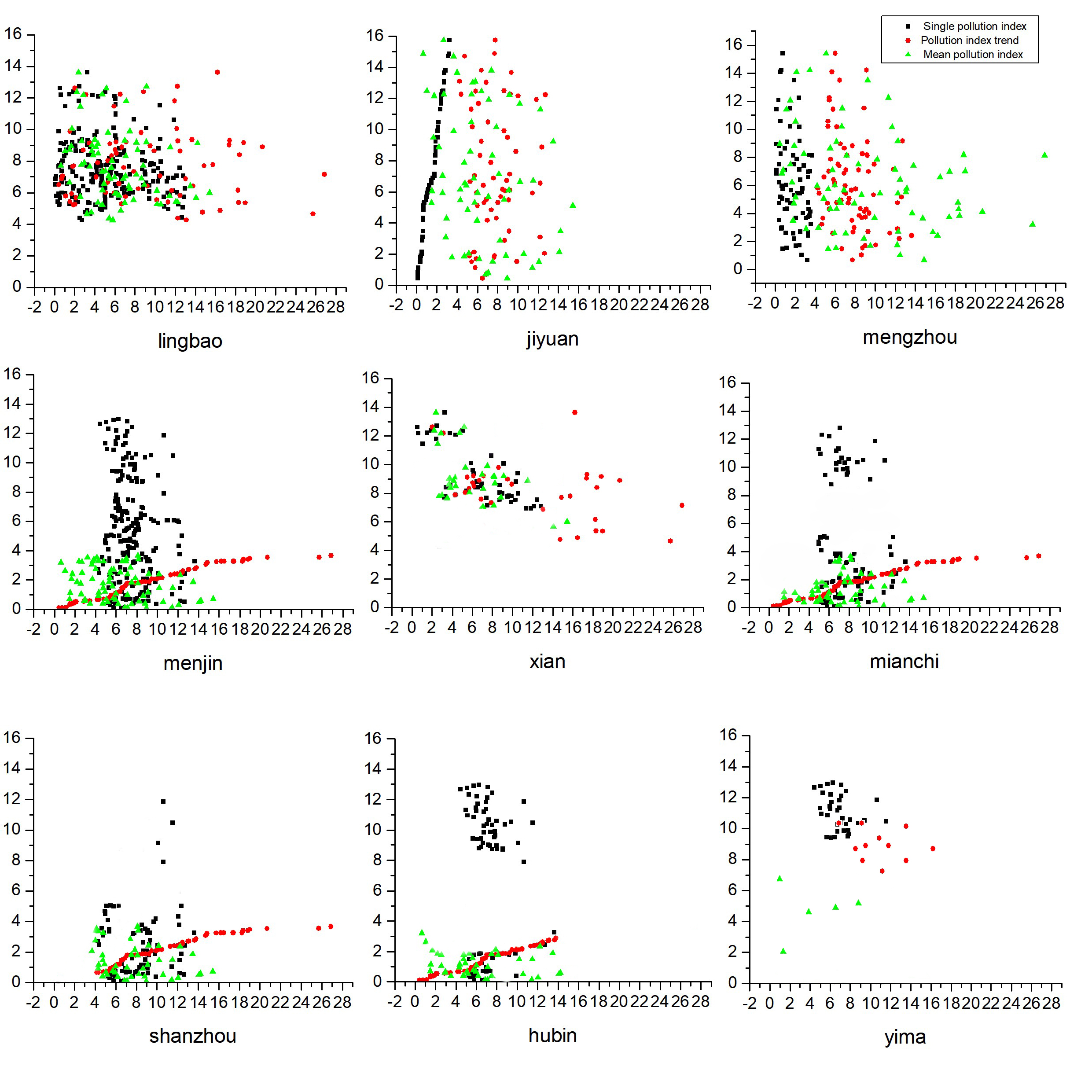

Supplement: Supplemental Information 1 [file peerj-11-16454-s001.zip › peerj-89657-peerj-89657-original_material_and_basic_data/Original Foundation Drawing/3 (1).jpg]

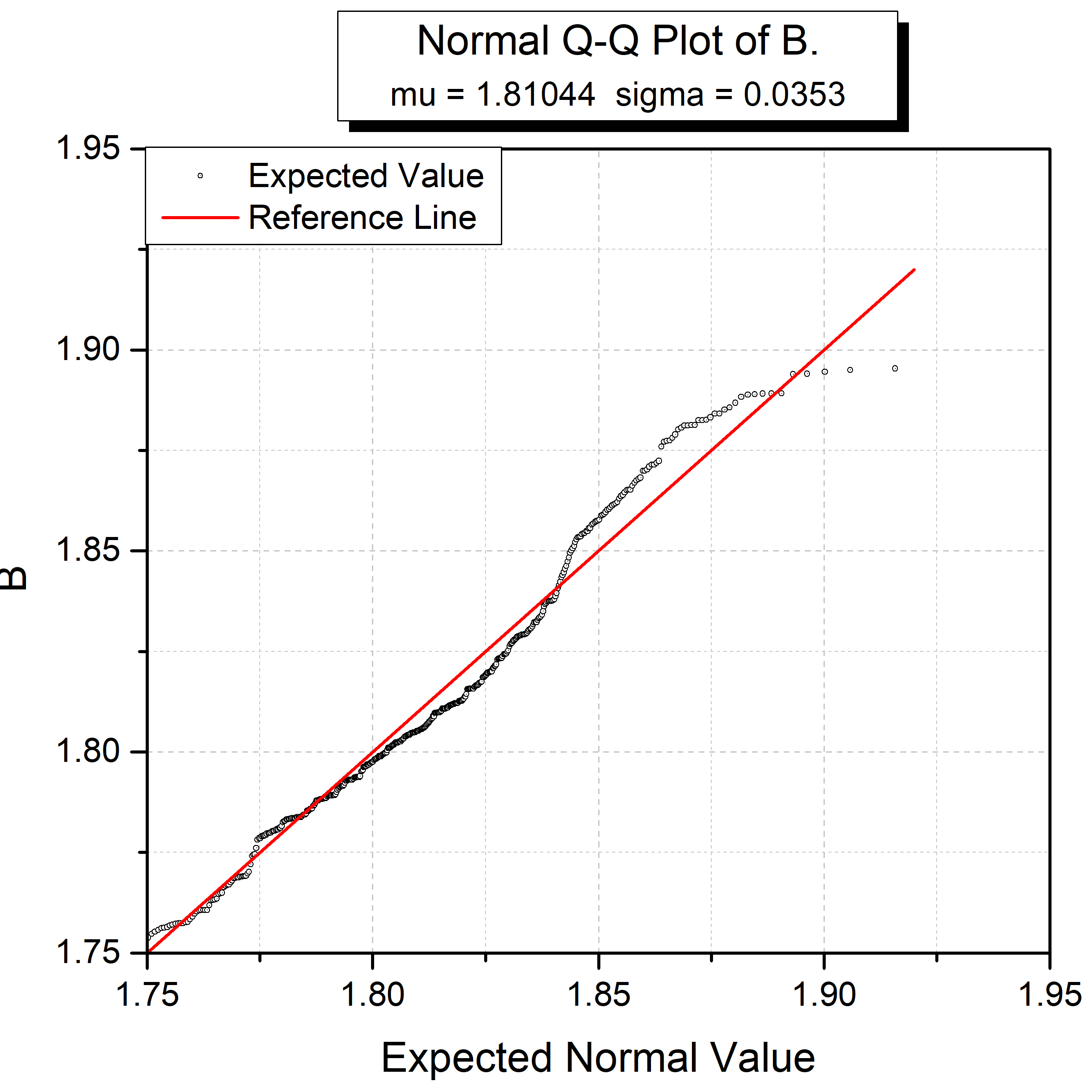

Supplement: Supplemental Information 1 [file peerj-11-16454-s001.zip › peerj-89657-peerj-89657-original_material_and_basic_data/Original Foundation Drawing/3 (1).png]

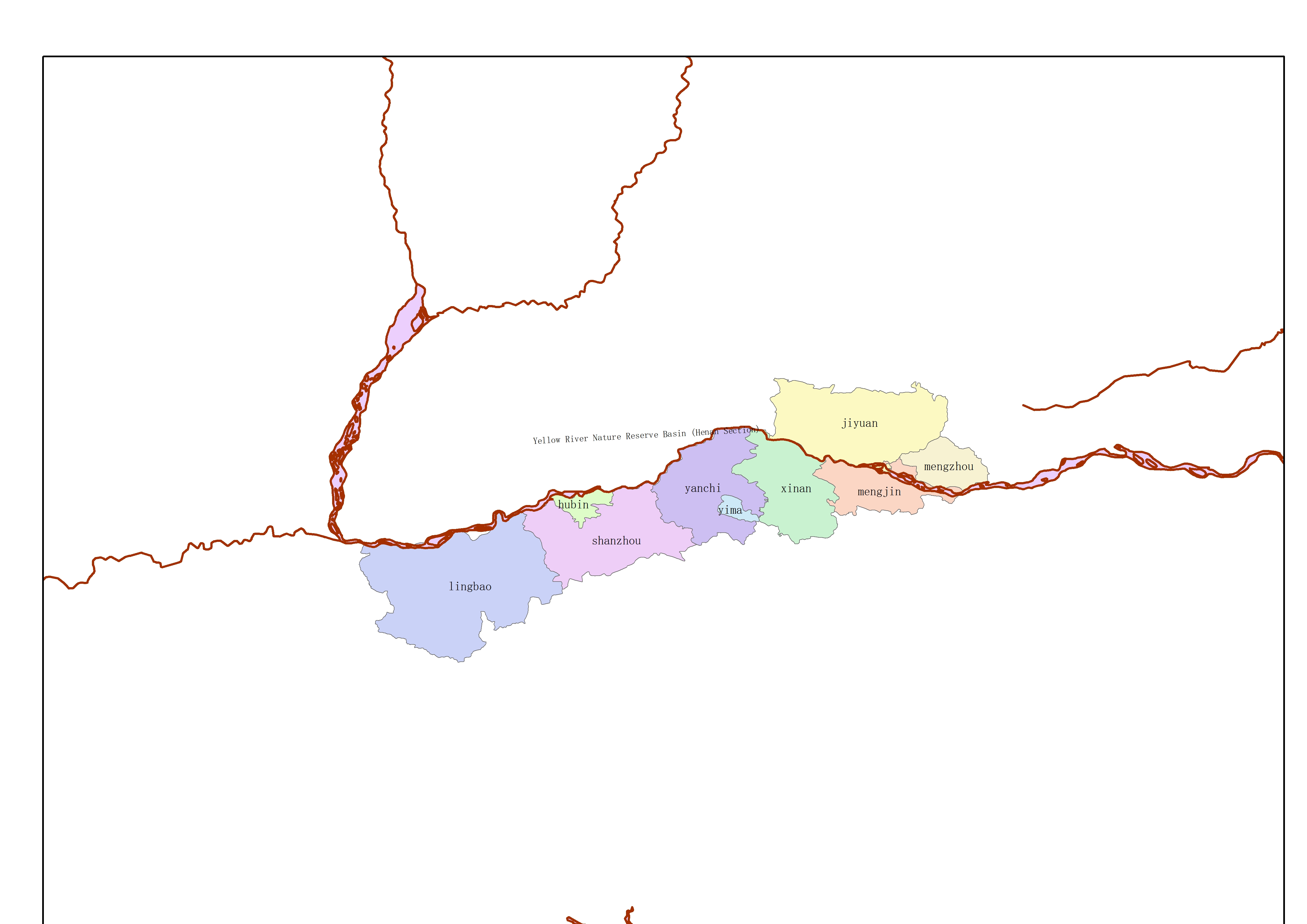

Supplement: Supplemental Information 1 [file peerj-11-16454-s001.zip › peerj-89657-peerj-89657-original_material_and_basic_data/Original Foundation Drawing/3 (2).jpg]

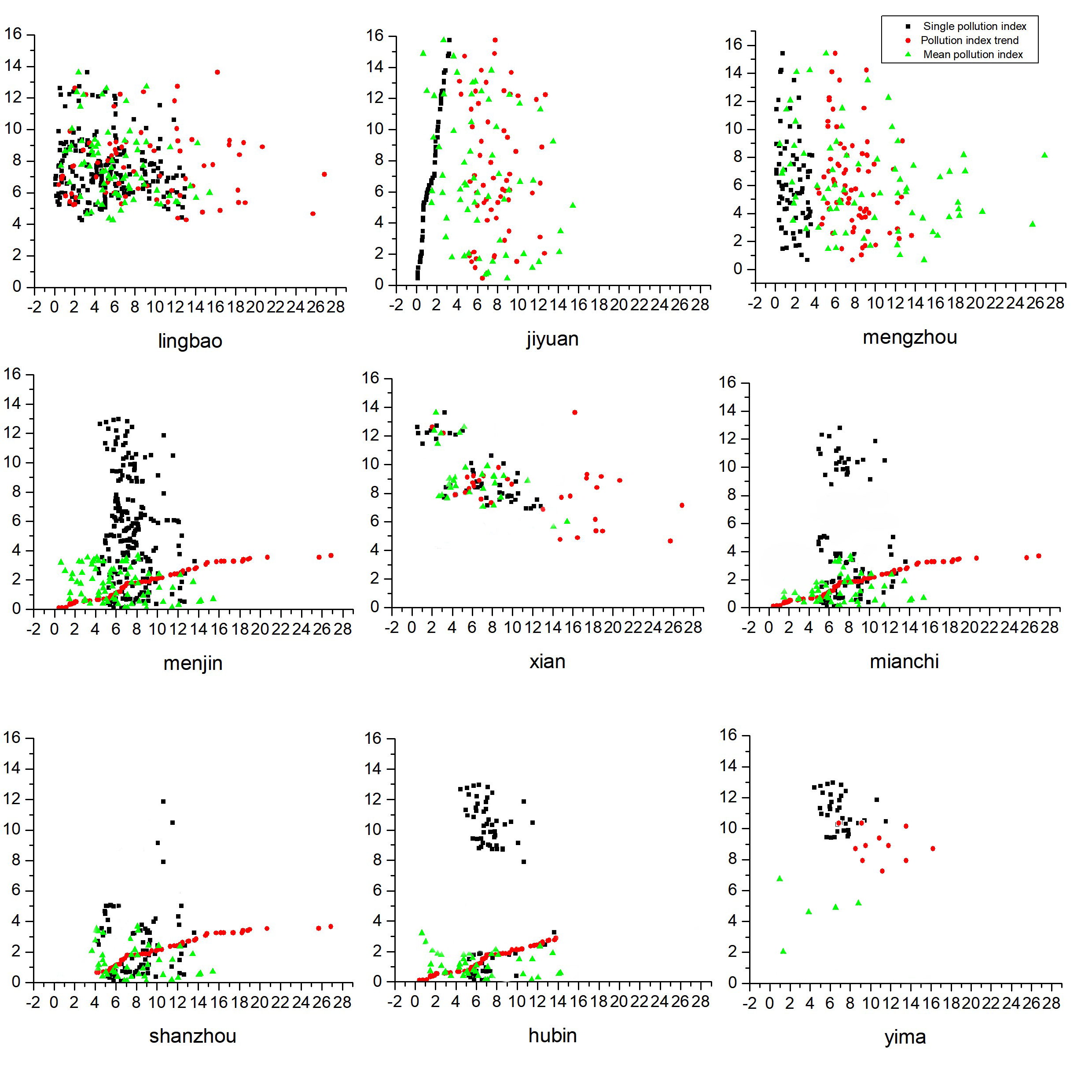

Supplement: Supplemental Information 1 [file peerj-11-16454-s001.zip › peerj-89657-peerj-89657-original_material_and_basic_data/Original Foundation Drawing/3 (2).png]

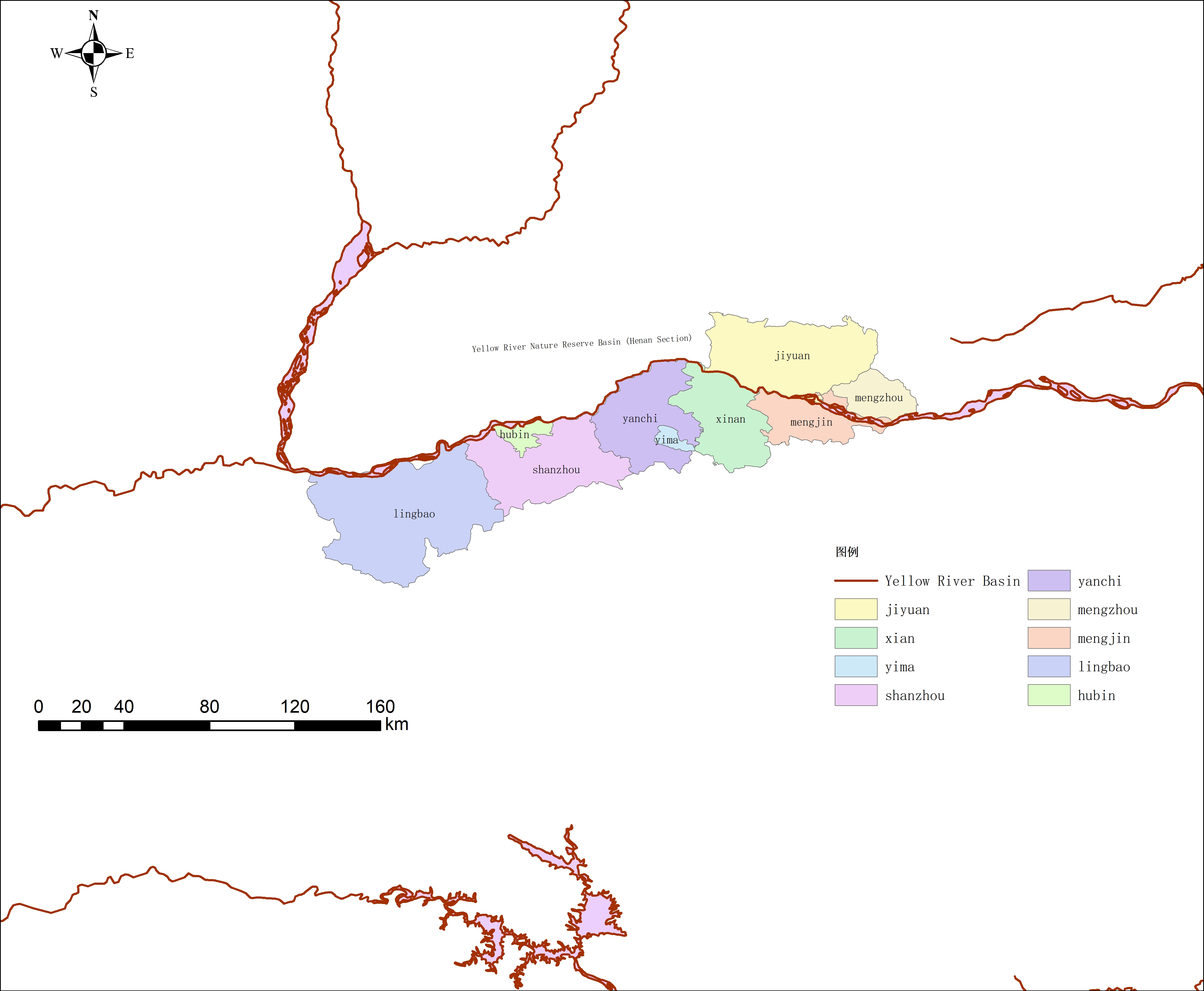

Supplement: Supplemental Information 1 [file peerj-11-16454-s001.zip › peerj-89657-peerj-89657-original_material_and_basic_data/Original Foundation Drawing/3 (3).jpg]

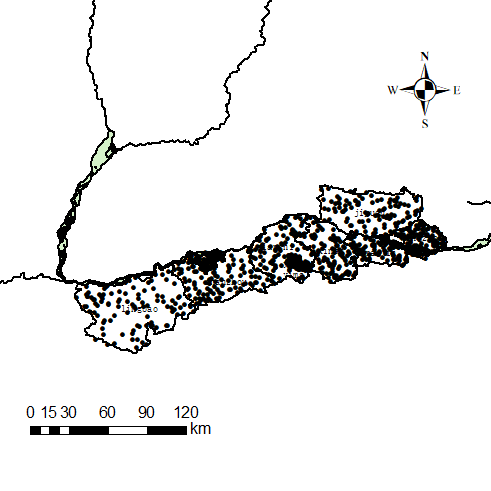

Supplement: Supplemental Information 1 [file peerj-11-16454-s001.zip › peerj-89657-peerj-89657-original_material_and_basic_data/Original Foundation Drawing/3 (3).png]

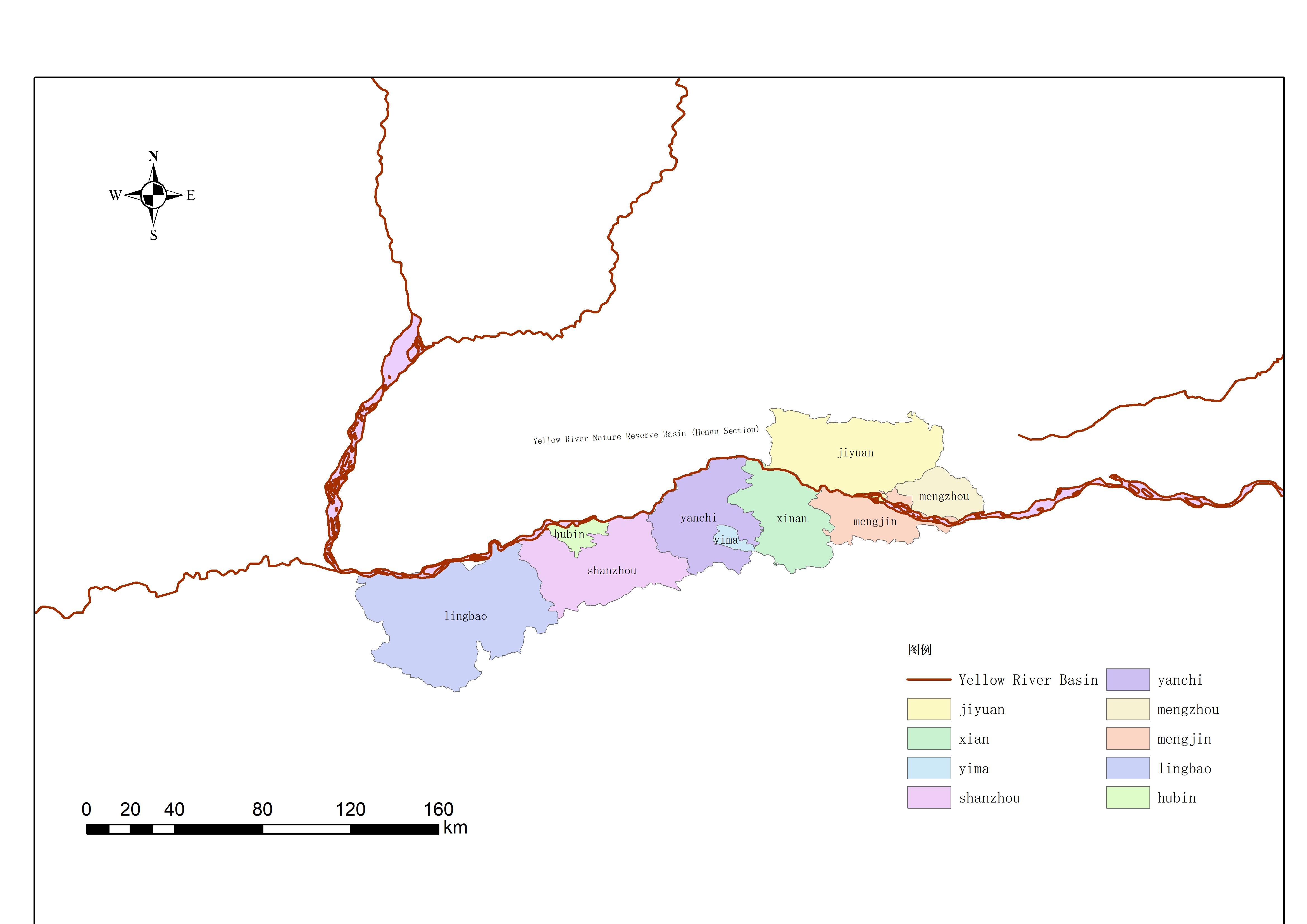

Supplement: Supplemental Information 1 [file peerj-11-16454-s001.zip › peerj-89657-peerj-89657-original_material_and_basic_data/Original Foundation Drawing/3 (4).jpg]

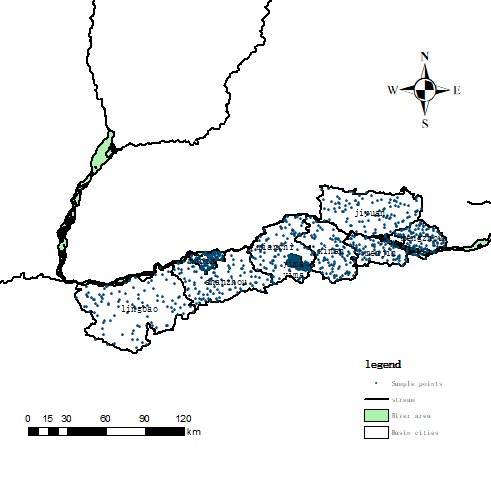

Supplement: Supplemental Information 1 [file peerj-11-16454-s001.zip › peerj-89657-peerj-89657-original_material_and_basic_data/Original Foundation Drawing/3 (4).png]

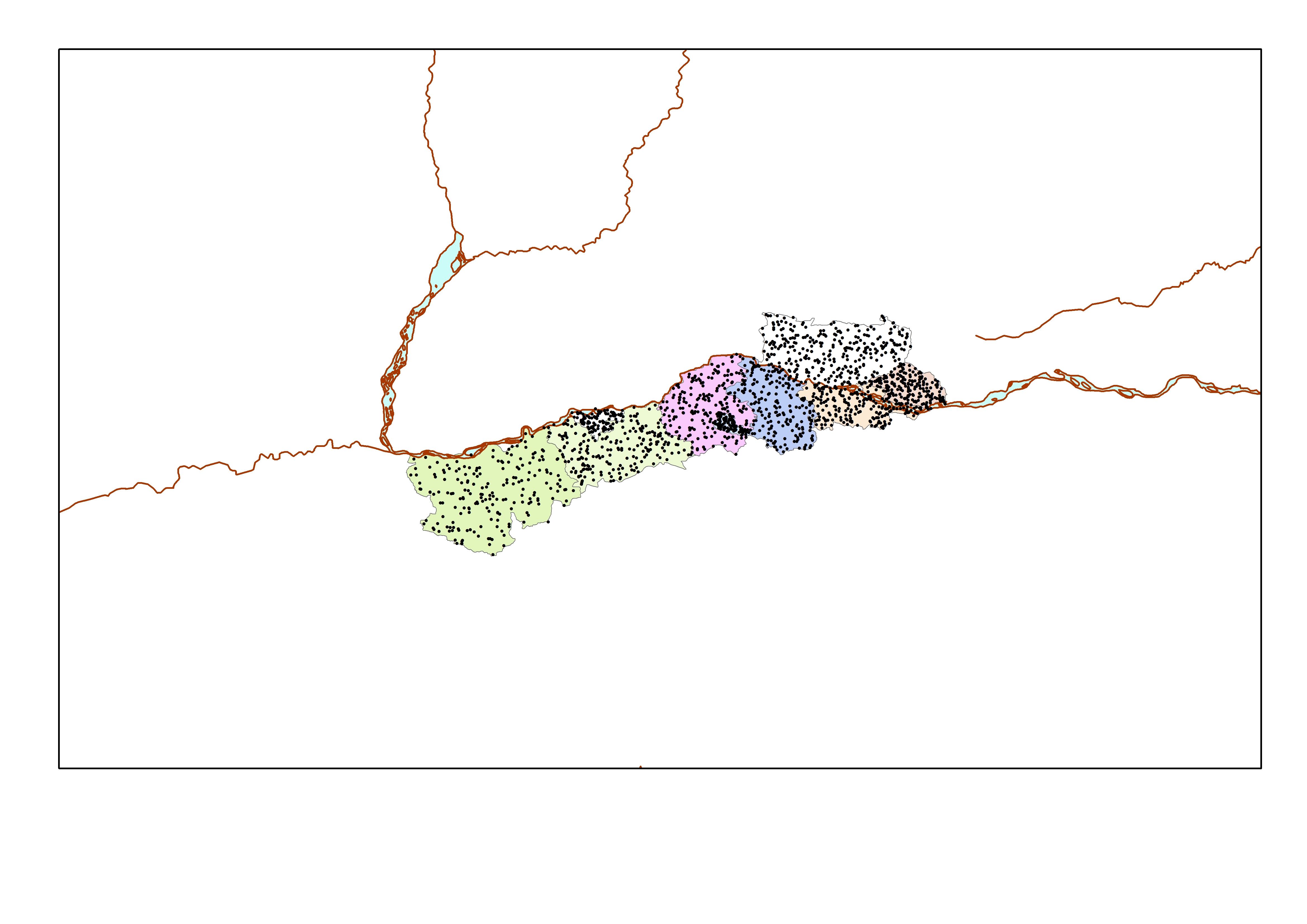

Supplement: Supplemental Information 1 [file peerj-11-16454-s001.zip › peerj-89657-peerj-89657-original_material_and_basic_data/Original Foundation Drawing/3 (5).jpg]

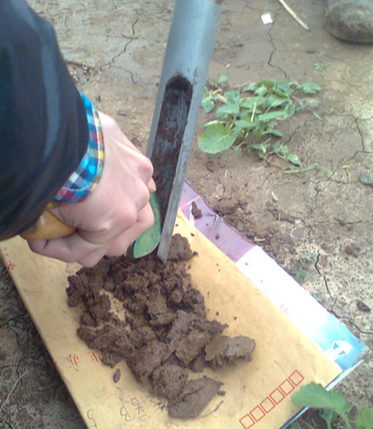

Supplement: Supplemental Information 1 [file peerj-11-16454-s001.zip › peerj-89657-peerj-89657-original_material_and_basic_data/Sample sampling site photos/1 (1).png]

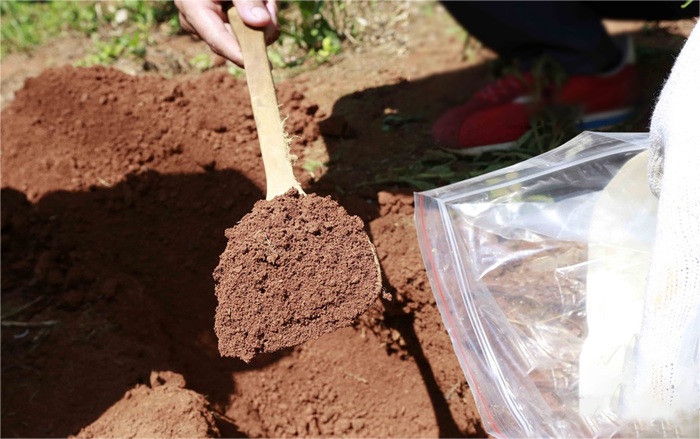

Supplement: Supplemental Information 1 [file peerj-11-16454-s001.zip › peerj-89657-peerj-89657-original_material_and_basic_data/Sample sampling site photos/1 (10).png]

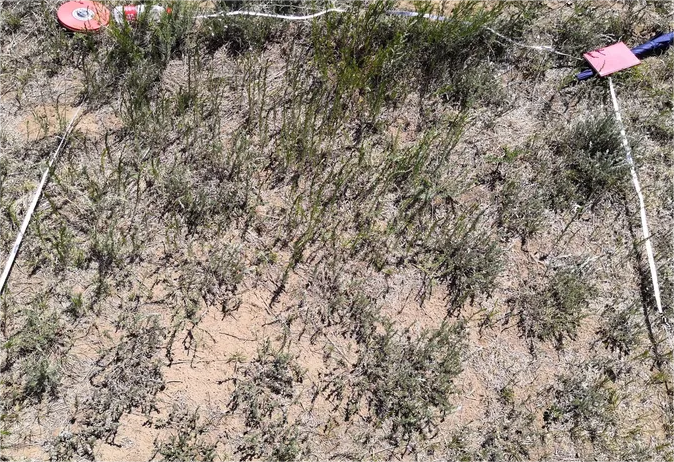

Supplement: Supplemental Information 1 [file peerj-11-16454-s001.zip › peerj-89657-peerj-89657-original_material_and_basic_data/Sample sampling site photos/1 (11).png]

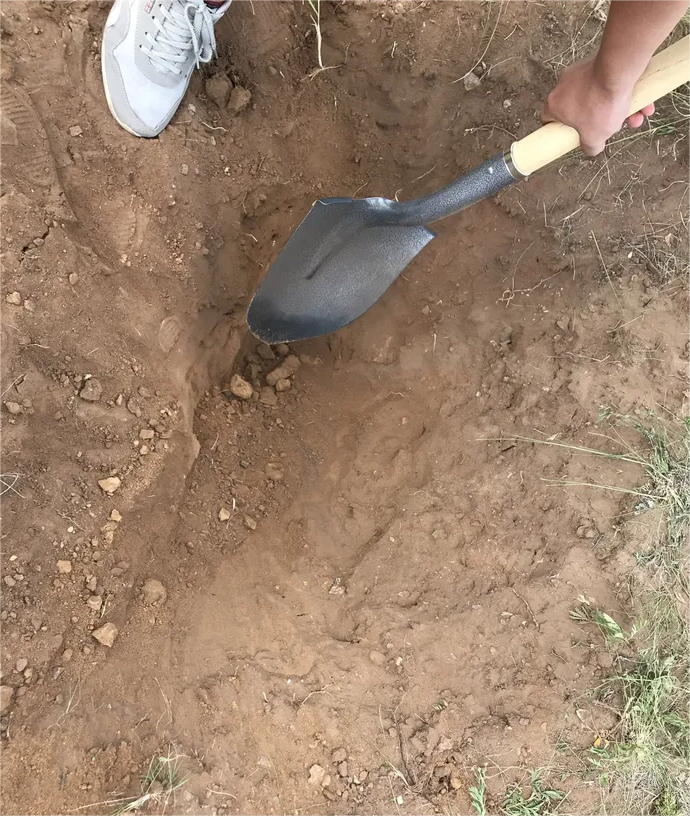

Supplement: Supplemental Information 1 [file peerj-11-16454-s001.zip › peerj-89657-peerj-89657-original_material_and_basic_data/Sample sampling site photos/1 (12).png]

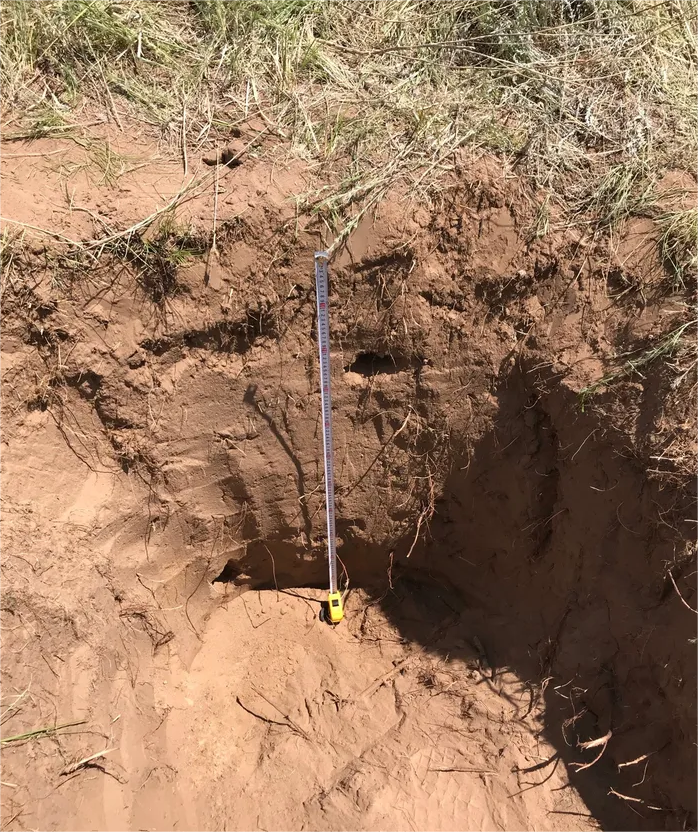

Supplement: Supplemental Information 1 [file peerj-11-16454-s001.zip › peerj-89657-peerj-89657-original_material_and_basic_data/Sample sampling site photos/1 (13).png]

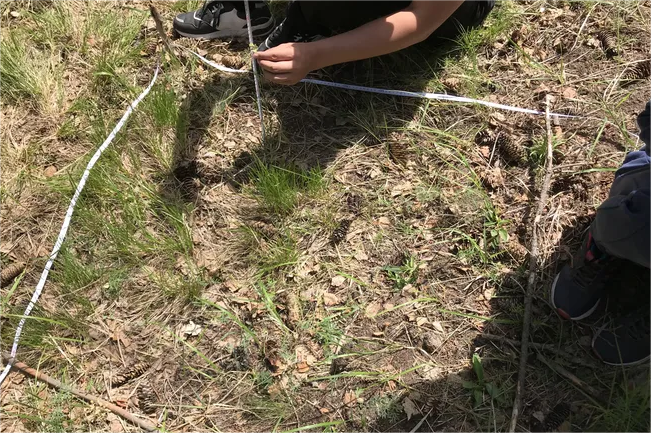

Supplement: Supplemental Information 1 [file peerj-11-16454-s001.zip › peerj-89657-peerj-89657-original_material_and_basic_data/Sample sampling site photos/1 (14).png]

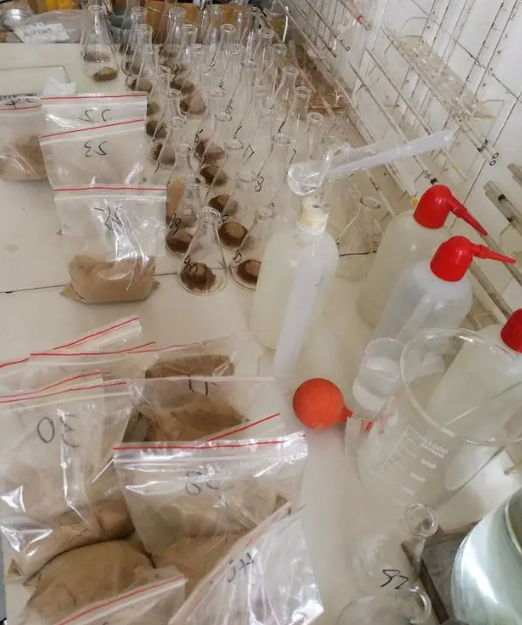

Supplement: Supplemental Information 1 [file peerj-11-16454-s001.zip › peerj-89657-peerj-89657-original_material_and_basic_data/Sample sampling site photos/1 (4).png]

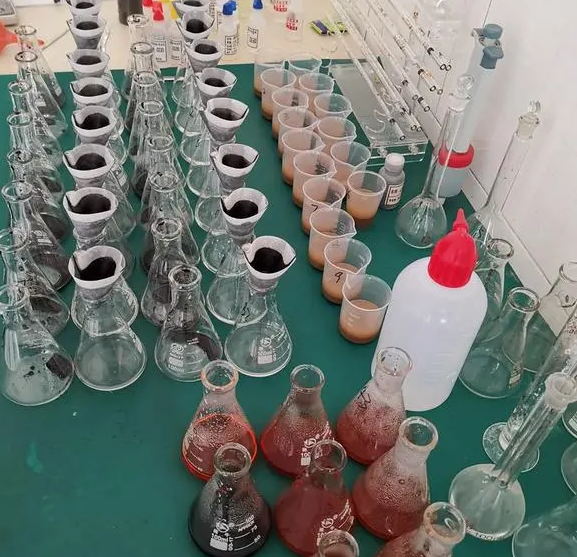

Supplement: Supplemental Information 1 [file peerj-11-16454-s001.zip › peerj-89657-peerj-89657-original_material_and_basic_data/Sample sampling site photos/1 (5).png]

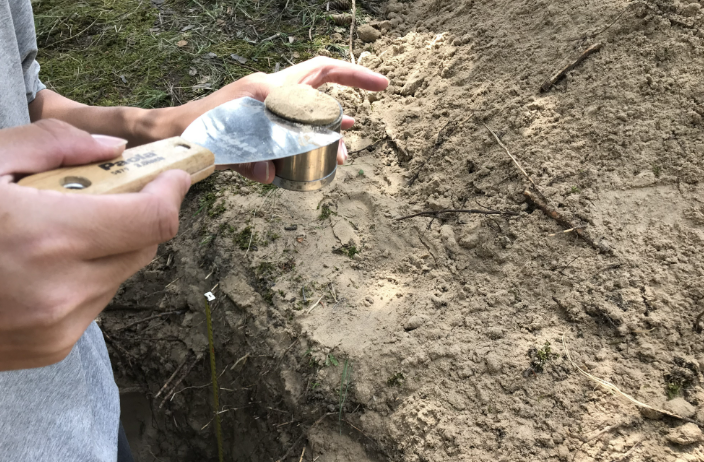

Supplement: Supplemental Information 1 [file peerj-11-16454-s001.zip › peerj-89657-peerj-89657-original_material_and_basic_data/Sample sampling site photos/1 (6).png]

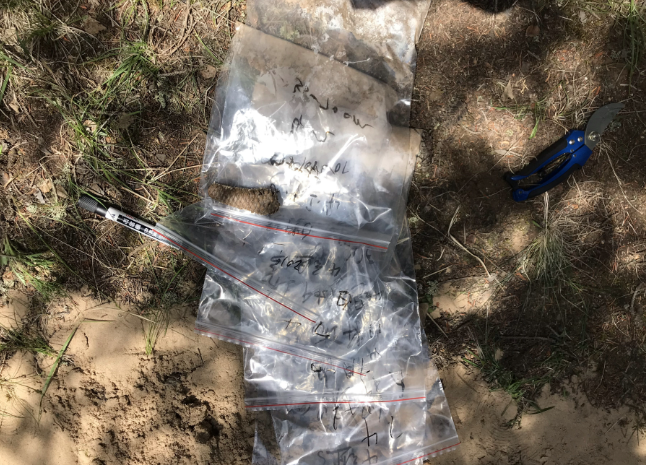

Supplement: Supplemental Information 1 [file peerj-11-16454-s001.zip › peerj-89657-peerj-89657-original_material_and_basic_data/Sample sampling site photos/1 (7).png]

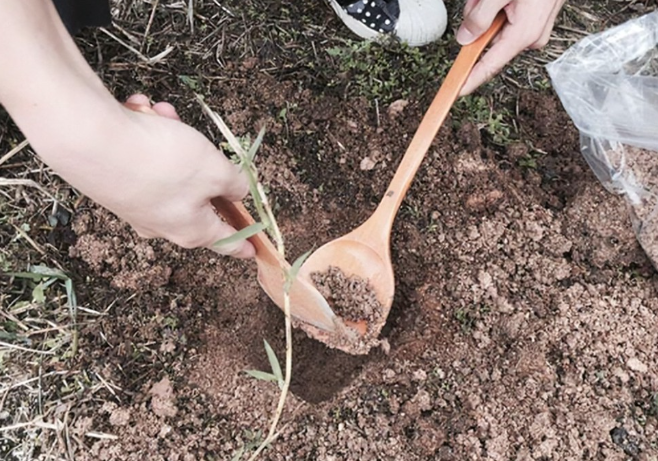

Supplement: Supplemental Information 1 [file peerj-11-16454-s001.zip › peerj-89657-peerj-89657-original_material_and_basic_data/Sample sampling site photos/1 (8).png]

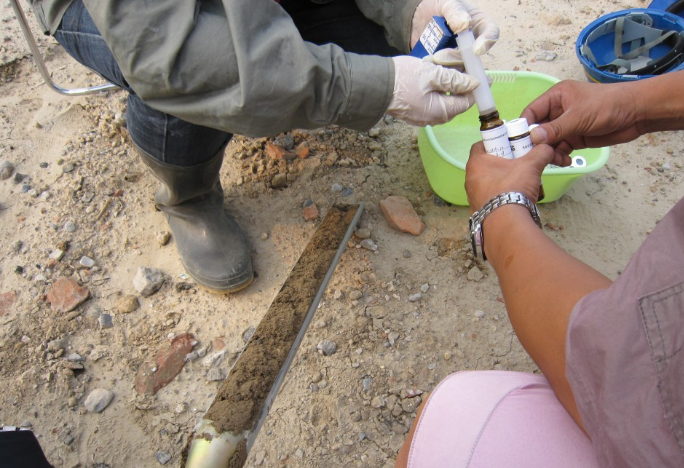

Supplement: Supplemental Information 1 [file peerj-11-16454-s001.zip › peerj-89657-peerj-89657-original_material_and_basic_data/Sample sampling site photos/1 (9).png]
